# Michigan State Board of Health

**Published:** 1878-09

**Authors:** 


### MICHIGAN STATE BOARD OF HEALTH.

The regular quarterly meeting of the Michigan State Board of Health occurred July 9, 1878, at Lansing, all the members being present.

Dr. Hitchcock offered the following resolutions, which were adopted:

*Resolved*, That this board respectfully request the board of regents of the University of Michigan and the trustees of the Detroit Medical College to establish in their respective institutions, at the earliest practicable moment, full chairs of public hygiene and fill the same with thoroughly competent professors.

*Resolved*, That this board respectfully request the controling board of all the collegiate institutions as well as the high-schools of the State to see that a course of instruction in public hygiene be given in each of their several institutions.

Dr. Kedzie presented some results of his investigations on the subject of lead-poisoning by means of tinned ware and other vessels containing lead.

He said it is well-known that there are substances actively poisonous when taken in large doses, that when taken in small but repeated doses often produce effects so obscure that they may be mistaken for the symptoms of some chronic disease. Lead, arsenic, antimony, and copper are examples.

The chronic poisoning which may be caused by minute doses of any of these metals, and the possibility of mistaking such metallic poisoning for some disease of a different nature, should warn us against their use, or make us careful and guarded while using them. Vessels in daily use for preparation or serving of food are especially liable to affect the physical condition if they contain any material which will insidiously sap the foundations of health and strength.

Culinary vessels which are cheap, durable, and convenient, and without injurious influences on the health, bear an important relation to the comfort and well-being of the people. Of all cheap metals for such use tin fulfills those conditions better than any other. It is compartively cheap, resists oxidation by exposure to air and water, has a white color, is not readily dissolved except by strong mineral acids, and the only salt of tin which is actively poisonous is the chloride, which will never be formed in the domestic use of tin vessels. The readiness with which iron surfaces may be coated over with it contributes to its valuable uses.

Unfortunately, while tin is comparatively cheap and safe, lead is cheaper and very dangerous. Yet the two metals readily unite forming an alloy which may be used in place of tin, but which will generally oxidize and be dissolved by acids more readily than either metal of which it is composed. The danger of poisoning by the use of such vessels is very great. The attention of the State board of health has been called to this subject by a letter from Dr. Dorsch of Monroe, who writes that he has seen cases of *paralysis agitans* which had been taken for chorea, although other symptoms of lead-poisoning were present, and investigation showed, in all cases, that cooking and eating with tin spoons or in earthen and iron vessels with a coat of lead were the cause. The same is true with milk vessels. The acid dissolved the lead salts, and children are poisoned, dying by tubercles of the brain, meningitis, fits, and paralytic affections.

Grown persons do not escape, although resisting longer. A similar danger arises from tea and coffee pots of earthern ware or composition metal, from tin sieves and funnels, and almost all cooking utensils used by the poor. They are about equally as dangerous as the adulteration of food and spices, so common all over the country.

The danger of lead-poisoning is a matter of great importance because so large a proportion of our population employ tinned vessels for culinary and table use. The alloy of tin and lead oxidizes much more readily than pure tin, and the oxide of lead is very soluble in acetic acid or vinegar, or lactic acid, forming sugar of lead. It also forms salts with malic and citric acids, which are contained in apples, cherries, gooseberries, currants, or any acid fruits. When cooked in vessels containing lead or even placed in them for some time, they are liable to take it up and become very injurious thereby, because all salts of lead are poisonous. In this way a large portion of our daily food may be a vehicle of poison if prepared or contained in vessels containing a sensible amount; and this danger is greater because the compounds of lead are cumulative in their influence. A person may not be poisoned by one or two small doses, but minute doses taken for a long time will break the health and even destroy life.

The doctor said that of a large number of specimens of tin plate, tinned iron, and other culinary articles examined by him, he found in almost every instance an alloy with lead, and it was often present in large quantities. It is an astonishing fact that a large proportion of the tinned wares in the market are unfit for use because of the large quantity of lead with which the tin is alloyed.

### TEST FOR LEAD.

Place a drop of strong nitric acid on the tinned surface and rub it over a space as large as a dime. Warm it very gently till dry, and then drop two drops of a solution of idodide of potassium on this spot. The bright yellow iodide of lead will form on the spot ir tin contains lead. This test can be rapidly applied and the results are decisive. The doctor was informed that a peculiar kind of tin plate, the tinning composed mostly if not entirely of lead, was coming into general use for roofing eave troughs, and water pipes. The lead thus exposed would be in conditions favorable for oxidation, and a quantity of oxide and carbonate of lead would be washed away in the rain water and deposited in the cistern with every storm. Susceptible persons may be poisoned by washing in such lead-charged water, and all persons drinking it even after it has been filtered will be in danger of chronic lead poisoning. Earthen vessels are usually glazed to overcome their porosity. In many cases this glazing consists of fusible silicates of the alkalies, and alkaline earths. These have no injurious influence on the health. Oxide of lead when added to the alkaline silicates, borates, etc., makes a very fusible and closely adhering glazing; and is sometime used, but its use is very dangerous, especially if the vessels contain acid substances. The glazing decomposes and lead salts form which either dissolve or are mechanically suspended in the contents of the jar, and there is great danger of chronic lead-poisoning. This danger is unfortunately very common.

### ENAMELED IRON VESSELS.

Within a short time an enamel has been successfully applied to vessels made of iron plate, the enamel or glazing taking the place of tin coating on tin plate. As these vessels are coming into general use it is a matter of public interest to know what would be their influence on public health. He said that a culinary vessel to be safe must be impermeable by water and grease. Metals, especially where vessels are made without seams or joints, such as pressed tinware, glass, and many kinds of porcelain, are admirable in this respect. Glazed crockery after the glazing is fissured is very poor in this respect. If the new enameled ware shall prove satisfactory it will be an important acquisition. At the present time the most hopeful outlook for good, safe, and cheap culinary vessels lies in the direction of some fixed unabsorbent enamel for pressed iron-ware which will maintain an unbroken surface under all conditions of domestic use.

Another indispensable condition for a safe culinary vessel is that it shall not contain any poisonus material by which the food cooked or contained in it, shall be injuriously affected.

The specimens of granite-ware which he had examined failed to reveal any poisonous or injurious substance. He regarded it as a safe material to use, but feared its power to resist the tendency to crack after it had been frequently heated. The marbleized iron-ware presented very different results. The enamel was found to contain a large amount of lead, and even traces of arsenic were obtained fron the enamel by the use of Marsh's apparatus. In a quart basin of this marbleized iron-ware he placed eight ounces of water containing five per cent of nitric acid, heated it boiling hot, and kept the whole in a warm place 24 hours, then evaporated the dilute acid to dryness, dissolved the residue in water, filtered, and from the filtrate precipitated the lead, obtaining in this way what was equivalent to 23 grains of lead. In a similar basin of marbleized iron-ware eight ounces of vinegar (free from lead) were placed and kept in a warm place 24 hours, and then treated in the same manner as the dilute acid. This resulted in obtaining what was equivalent to seven grains of lead. On powdering some of of the enamel and heating it with concentrated acids, very distinct traces of arsenic were obtained. This was probably not present by design, but accidentally from being contained in some of the substances used in making the enamel. A culinary vessel which contains so much lead, and in such a state of feeble combination that eight ounces of ordinary cider vinegar can in 24 hours dissolve from a quart basin what is equivalent to seven grains of metallic lead, must be a very unsafe vessel for general use.

